# Effect of Intercritical Temperature on the Microstructure and Mechanical Properties of a Ferritic–Martensitic Dual-Phase Low-Alloy Steel with Varying Nickel Content

**DOI:** 10.3390/ma15249018

**Published:** 2022-12-16

**Authors:** Esteban Rodoni, Kim Verbeken, Tom Depover, Mariano Iannuzzi

**Affiliations:** 1 Curtin Corrosion Centre, Curtin University, Perth 6102, Australia; 2 Research Group Sustainable Materials Science, Department of Materials, Textiles and Chemical Engineering, Ghent University, 9000 Ghent, Belgium

**Keywords:** dual-phase steel, intercritical annealing, mechanical properties, microstructure characterization

## Abstract

Dual-phase low-alloy steels combine a soft ferrite phase with a hard martensite phase to create desirable properties in terms of strength and ductility. Nickel additions to dual-phase low-alloy steels can increase the yield strength further and lower the transformation temperatures, allowing for microstructure refining. Determining the correct intercritical annealing temperature as a function of nickel content is paramount, as it defines the microstructure ratio between ferrite and martensite. Likewise, quantifying the influence of nickel on the intercritical temperature and its synergistic effect with the microstructure ratio on mechanical properties is vital to designing dual-phase steels suitable for corrosive oil and gas services as well as hydrogen transport and storage applications. In this work, we used a microstructural design to develop intercritical annealing heat treatments to obtain dual-phase ferritic–martensitic low-alloy steels. The intercritical annealing and tempering temperatures and times were targeted to achieve three different martensite volume fractions as a function of nickel content, with a nominal content varying between 0, 1, and 3-wt% Ni. Mechanical properties were characterized using tensile testing and microhardness measurements. Additionally, the microstructure was studied using scanning electron microscopy coupled with electron backscatter diffraction analysis. Tensile strength increased with increasing martensite ratio and nickel content, with a further grain refinement effect found in the 3-wt% Ni steel. The optimal heat treatment parameters for oil and gas and hydrogen transport applications are discussed.

## 1. Introduction

Infrastructure operating in hydrogen-containing environments or that produced hydrogen as a consequence of cathodic protection or as a result of corrosion are often prone to deterioration. The degradation phenomena associated with hydrogen is known as hydrogen embrittlement (HE), which reduces the ductility of susceptible materials, leading to fast brittle failure [1]. For hydrogen-related applications where hydrogen is used as an energy carrier, such as hydrogen transport and storage, atomic hydrogen is sourced by the dissociation of gas molecules. Therefore, although hydrogen as an energy carrier is a potential and promising sustainable solution to fossil fuels [2], the appropriate material selection is challenging [3]. In addition, the lack of available data on hydrogen transport results in conservative design codes increasing the costs associated with hydrogen pipelines and reducing their cost competitiveness [4]. HE is also a common failure mode in oil and gas (O–G) applications, where hydrogen is present due to electrochemical reactions, e.g., the cathodic reaction associated with corrosion and as a result of cathodic protection. In the case of O–G fields producing H_2_S, i.e., sour environments [5], a particular and extreme HE mode, referred to as sulfide stress cracking (SSC), has been associated with catastrophic failures [6]. Therefore, materials with a tailored composition and microstructure are paramount to satisfy the increasing demands of the hydrogen and O–G sectors.

Low-alloy steels (LAS) are widely used in O–G production due to their excellent mechanical properties and low cost. High strength and adequate toughness can be achieved through thickness when combining chemical composition with a correct heat treatment process. Elements such as chromium, molybdenum, manganese, and nickel (Ni) are commonly added to LAS to improve mechanical and technological properties. Ni is the only element that increases strength and fracture toughness simultaneously, increases hardenability, and lowers the ductile to brittle transition temperature (DBTT) while having a low penalty on weldability [7]. Ni remains in solid solution up to 7-wt% Ni [8] and stabilizes the austenite phase, suppressing the transformation temperatures A_c1_ and A_c3_.

The ASME B31.12 [3] code regulates LASs for hydrogen piping and pipelines. The standard limits the allowable Ni nominal content to a maximum of 0.5-wt%, despite Ni’s beneficial effects. Similarly, ISO 15156-2 [9]—i.e., the international standard regulating the use of low alloy steels in source service—restricts the allowable Ni content to a maximum of 1-wt% Ni on LAS in environments containing H_2_S. Alloys that do not satisfy the standard’s criteria must undergo a strict qualification process; consequently, alloys with outstanding performance in other industries are excluded in practice. The cap on nickel stated by the ISO 15156-2 standard [9] is based on the research by Treseder and Swanson [10], which was soon questioned by Snape [11] through an extensive correlation study between LAS strength, microstructure, and failure mechanism. Snape argued that alloys, unintentionally tempered above A_c1_, would promote the formation of untempered martensite. As reviewed by Craig [8] and Kappes et al. [7] and reported by others [12,13,14], SSC susceptibility is related to a complex interplay between the microstructure, chemical composition, impurities, and thermo-mechanical processes rather than the sole effect of Ni metallurgical composition. As shown by Snape, a microstructure composed of tempered martensite with a uniform distribution of fine spheroidized carbides on a ferritic matrix exhibited the best SSC performance. Conversely, the presence of untempered martensite had a detrimental effect on the cracking resistance which implies that retained austenite, as a potential source of untempered martensite, should be reduced [11]. Therefore, the focus should be placed on the heat treatment process, as it alters the material’s microstructure and its HE resistance.

Intercritical annealing is one of the most common heat treatments to produce ferritic–martensitic dual-phase (DP) steels [15], which involves heating the microstructure into the intercritical annealing range to obtain a target austenite-to-ferrite ratio. The austenite-ferrite ratio is determined based on the set temperature, followed by a quenching step transforming austenite into martensite [15]. Snape et al. [16] modified commercial LASs using intercritical annealing to evaluate the SSC performance of DP-LAS with varying Ni contents. The author reported that the LAS with the highest Ni content (i.e., SAE 4340, 1.82-wt% Ni), with a yield strength of 737 MPa (107 ksi), was immune to SSC. Snape et al. attributed the outstanding performance of the DP-LAS 4340 over the Ni-free LASs to the effect on Ni in lowering the A_c1_, thus, allowing intercritical annealing at a lower temperature and the corresponding carbide refinement.

DP-LASs are extensively used in the automobile industry as their microstructure combines a ductile soft ferrite matrix that provides high formability with a high strength untempered martensitic phase [15]. Despite the advanced properties of the DP high-strength low-alloy (HSLA) steels designed for the car industry, many authors have stated their poor HE resistance [17,18,19,20]. The poor HE resistance of automotive DP-LASs is due to the presence of untempered martensite, which is highly deleterious. Tempering is often suggested to decrease HE susceptibility [19,21,22].

The advantages offered by the DP microstructures used in the automotive and nuclear industries make ferritic–martensitic DP-LASs promising candidates for hydrogen-related applications and sour O–G service. Nevertheless, new heat treatments must be designed to tailor microstructures that meet HE resistance requirements. This work focuses on the microstructure design of DP-LASs with incremental nickel concentrations produced by intercritical annealing and tempering. The resulting DP-LASs are characterized as a function of varying nickel content. This study is the first step of an extensive program to determine the effect of microstructure and Ni alloying on the HE resistance of DP-LASs.

## 2. Materials and Methods

### 2.1. Materials

For this study, the manufacturing process was tailored to reduce segregation and achieve a homogeneous composition through-thickness. The nominal Ni content was varied between 0, 1, and 3-wt% Ni while keeping the rest of the alloying elements fixed. The actual composition of these LASs is shown in Table 1. The research-grade LASs were vacuum melted at 1600 °C in alumina crucibles and then poured into a mold to produce 203.2 mm × 304.8 mm × 76.2 mm ingots. Afterwards, the ingots were soaked at 1150 °C for 3 h before rolling into 254 mm wide and 23.5 mm thick plates. Finally, the plates were flattened, de-scaled, and cut from the core to their final dimensions of 100 mm width, 130 mm length, and 10 mm thickness. The microstructure of the as-received material comprised a ferrite-pearlite structure with varying degrees of banding, as discussed below.

### 2.2. Heat Treatment Design

**Figure 1:**
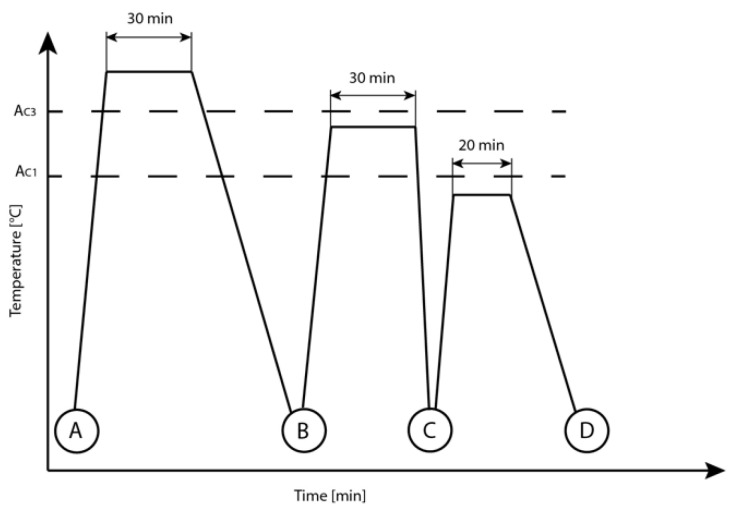
Schematic representation of the heat treatment stages: Full annealing (A,B), intercritical annealing and quenching (B,C), and tempering (C,D).

The heat treatment consisted of three stages, as described in Figure 1. First, a full annealing stage for 30 min at 900 °C followed by furnace cooling, aimed to reduce the preferential grain direction due to hot rolling. Second, intercritical annealing for 30 min followed by quenching in ice water defined the microstructure ratio to produce the desired amount of untempered martensite and ferrite. Finally, a tempering stage tempered the martensite, precipitating fine carbides on the martensite laths and reducing the hardness of the DP-LASs.

The ferrite and austenite (α + γ) temperature range (between A_c1_ and A_c3_) was determined using the empirical expressions given by Andrews in Equations (1) and (2), which defined the intercritical heating temperatures [25]. The critical temperatures for this batch of research-grade LASs were obtained by Parodi et al. [26] using differential scanning calorimetry. Ten holding temperatures were selected from the (α + γ) region to target the intercritical temperatures to obtain the desired microstructure ratios.

Ac_1_ = 723 − 10.7 Mn − 16.9 Ni + 29.1 Si + 16.9 Cr + 290 As + 6.38 W (1)

Ac_3_= 910 − 203 √C − 15.2 Ni + 44.7 Si + 104 V + 31.5 Mo + 13.1 W (2)

### 2.3. Microstructure Characterisation

For the phase identification, heat-treated samples that were 8 mm long, 5 mm wide, and 10 mm thick were cut in halves to expose the bulk condition, therefore, avoiding the zone affected by decarburization. Samples were mounted in epoxy resin and cured before grinding and polishing. Samples were then ground using sequentially finer SiC paper from P320, P600, P1200, and P5000. Ground samples were finally polished with 3 and 1 μm particle size diamond suspensions. After polishing, samples were etched in Nital 2% for 40 s to reveal the phases. Phase volume fractions were determined by point counting following the ASTM E562-19 [27]. For each sample, 100 micrographs taken using scanning electron microscopy (SEM) with a view field of 250 μm were analyzed in Image J software using a 25-points grid.

Grain size characterization was performed by electron backscatter diffraction (EBSD) on performed slow strain rate test (SSRT) samples with a region of interest set to 2.5 × 2.5 mm with a step size of 2.4 μm. In addition to the grinding and polishing steps described above, the specimens were subjected to chemical-mechanical polishing using colloidal silica with 0.04 μm, and ion milling to reduce the effect of plastic deformation resulting from grinding. Samples were carbon-coated before EBSD analysis using a sputtering magnetron to reduce drifting during acquisition. Data analysis was performed using the Aztec Crystal software with a threshold of 10° and a minimum area of 10 pixels. Statistics were based on equivalent circle diameter, following the ASTM E2627 standard [28]. Due to the low index quality of the martensite phase, the grain size analysis only considers the ferritic matrix.

### 2.4. Quantification of Retained Austenite by XRD

XRD analysis was performed to quantify the amount of face-centered cubic (FCC) phase due to the potentially detrimental effect of retained austenite on HE resistance. The selected sample had the highest Ni content (3-wt% Ni) as Ni stabilizes austenite, making this sample the most prone to a higher retained austenite content and, hence, providing conservative results. A Cobalt target was used with 35.0 kV voltage and 40.0 mA current for the XRD measurements.

### 2.5. Mechanical Characterisation and Fractography

Hardness tests were performed to evaluate the steel properties before and after tempering to meet the 250-HV (22-HRC) hardness restriction of the ASTM B31.12 and ISO 15156-2 standard. For this analysis, samples were randomly indented in 25 points with a load of 1 kgf according to the E384-17 standard [29].

**Figure 2:**
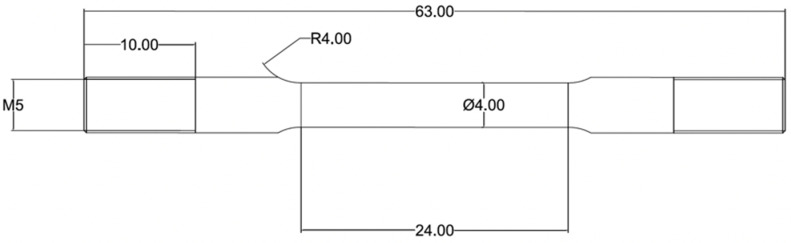
SSRT sample dimensions. All numbers in mm.

For the tensile tests, the as-received plates for each Ni nominal content were cut into three bars, one for each microstructure ratio, and then heat treated to obtain the desired microstructure. After heat treatment, tensile test samples were extracted along the rolling direction by electric discharge machining (EDM) and machined in a lathe to their final dimensions following the ASTM E8M standard [30]. The sub-size tensile sample dimensions are shown in Figure 2. The samples’ surface finish was obtained using successively finer SiC sandpaper from P320, P600, P1200, and P5000 in the tangential direction. Samples were ground parallel to the gauge section with P5000 SiC grit size to reduce the machining and grinding marks perpendicular to the tensile applied load. The strain rate was set to 10^−6^ s^−1^ (slow strain rate), as the results obtained in this work will be used as the baseline for future HE evaluations. Engineering stress–strain curves were analyzed following the standard [30]. Although displacements were obtained from the cross-head movement, the integration of the stress–strain curve (i.e., the area under the curve) was used as an indirect measurement of the absorbed energy during the tensile test, and in the present work, is described as toughness [31]. This analysis is a relative comparison between different sample conditions tested with the same machine.

After mechanical testing, the tensile samples’ gauge sections were analyzed using SEM to evaluate the fracture surface to determine the fracture mode and characterize potential secondary cracking. In addition, a fracture cross-section was analyzed by SEM-EBSD to evaluate the crack growth path.

## 3. Results

### 3.1. Microstructure Characterisation

Micrographs of the heat-treated samples obtained from the different heat treatment stages are provided in the Supplementary Figure S1. The as-received plates presented a ferritic–pearlitic microstructure with severe banding and grains elongated along the rolling direction, as shown in Supplementary Figure S1A. The microstructure obtained after full annealing for 30 min at 900 °C and cooling in the furnace, shown in Supplementary Figure S1B, exhibited a more equiaxial grain distribution while minimizing grain growth. The intercritical annealing for 30 min and quenching in ice water resulted in a DP ferritic–martensitic microstructure, as shown in Supplementary Figure S1C. Lastly, samples tempered at 550 °C revealed a microstructure composed of tempered martensite and ferrite (Supplementary Figure S1D).

**Figure 3:**
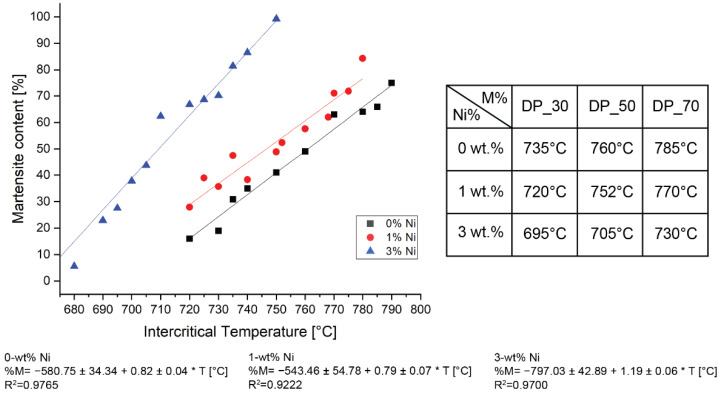
Martensite content from samples heat-treated for 30 min at varying intercritical temperatures. Analysis of martensite composition was performed by point counting following the ASTM E562-19 [27].

Figure 3 shows the martensite percentage as a function of the intercritical temperatures. Additionally, representative micrographs of the varying intercritical temperatures and Ni contents are shown in the Supplementary Figure S2. At higher holding temperatures, the austenite volume increased, subsequently transforming into martensite by quenching. Martensite increased approximately linearly with increasing intercritical temperature for a given Ni composition. Three microstructure ratios were targeted for each Ni composition based on the correlation between intercritical temperatures and the obtained martensite contents. For the rest of the work, samples whose martensite content was not confirmed by SEM are noted as DP_30%, DP_50%, and DP_70% based on the intercritical annealing temperatures to obtain 30, 50, and 70 per cent of martensite, respectively.

**Figure 4:**
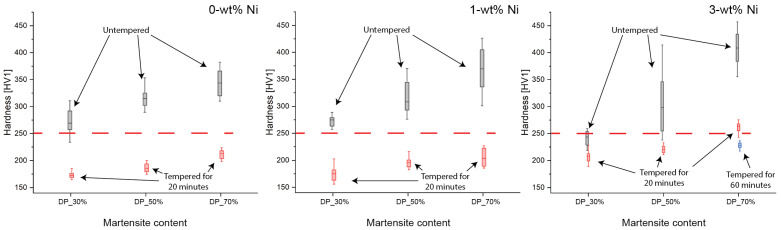
Hardness values for 0, 1, and 3-wt% Ni with DP_30%, DP_50%, and DP_70% in the as-quench and tempered condition plotted as black and red boxes, respectively. Tempered samples were heat treated at 550 °C for 20 min, and for 3-wt% Ni DP_70%, the tempering time was extended to 60 min (blue box) to meet the ASME B31.12 [3] and ISO 15156-2 [9] hardness limit, indicated as a red dash line.

Figure 4 summarizes the hardness results for samples in the as-quenched condition and tempered condition categorized by martensite content for 0, 1, and 3-wt% Ni. When analyzing the samples in the as-quenched condition, the increased martensite content increased the hardness value irrespective of Ni content. As a result, most quenched samples exhibited hardness values over 250 HV, regardless of the microstructure ratio and chemical composition. As described in the Introduction, this work intends to meet the ASME B31.12 [3] and ISO 15156-2 [9] standards, which limits the hardness to 250 HV; therefore, a tempering stage was required to temper the fresh, untempered martensite and reduce its hardness and improve HE resistance [7,11].

Figure 4 also compares the hardness values distribution obtained after the tempering stage (red boxes). Most samples met the hardness limit, plotted in red dash line, after tempering for 20 min. Only for the sample with the highest Ni and martensite contents (i.e., 3-wt% Ni DP_70%) the tempering time was extended to 60 min to meet the threshold (blue box).

**Figure 5:**
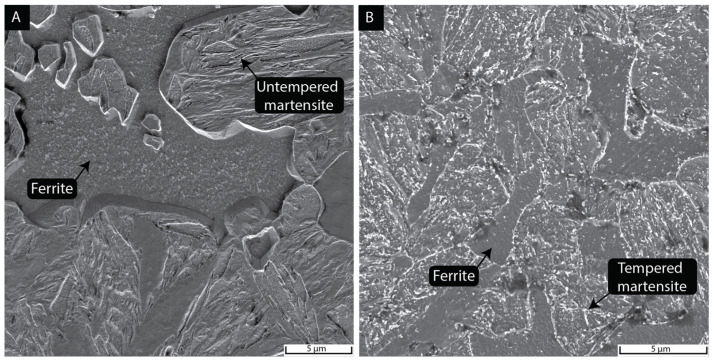
Scanning electron micrographs of 1-wt% Ni DP_70% in the as-quenched condition (**A**) and after temper (**B**). Untempered martensite displays a needle and rough appearance while ferrite is present as a smooth area. Tempered microstructure displayed carbides at boundaries and within martensitic laths.

Changes in microstructure due to the tempering step were also visible in the SEM micrographs comparing untempered and tempered conditions. For example, Figure 5A show the microstructure of a 1-wt% Ni DP_70% sample before tempering, with a ferritic phase (smooth surface) and untempered martensite (needle/blocky shape). The presence of carbides in the ferritic region could be attributed to undissolved carbides from the intercritical range [32]. During tempering, martensite released the carbon supersaturation by carbide precipitation, i.e., cementite [33]. The tempered microstructures Figure 5B had carbides that ranged between 30–400 nm in size, mainly located in the boundaries and within the previous martensitic laths (Figure S3B).

### 3.2. Quantification of Retained Austenite by XRD

**Figure 6:**
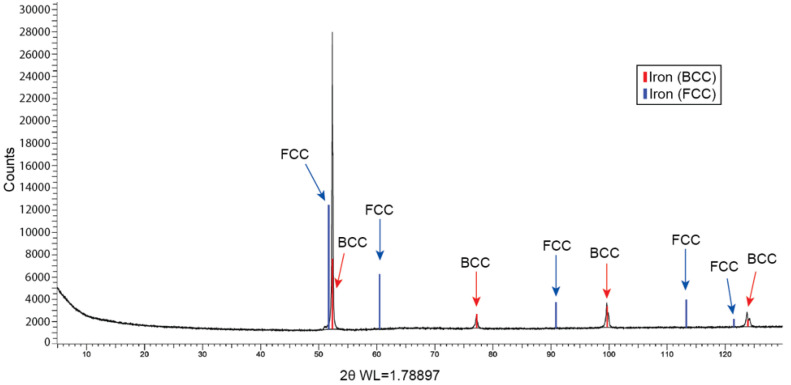
XRD analysis on sample 3-wt% Ni DP_70%. Red and blue lines indicate the angles corresponding to BCC (ferrite or martensite) and FCC (retained austenite), respectively. The absence of peaks matching with blue lines indicates that no retained austenite was found.

Figure 6 illustrates the diffraction pattern of the 3-wt% Ni DP_70% sample, in which the characteristic patterns for body-centered cubic (BCC) and FCC are displayed in red and blue, respectively, and labelled. The peaks matching red lines corresponding to BCC crystal structures represent ferritic or martensitic phases. In addition, if present, peaks associated with blue lines would indicate austenite. The implemented method revealed diffracted XRD peaks correlated to crystallographic phases with a concentration higher than 1-wt%. XRD peaks associated with FCC were not found, indicating the absence or a concentration lower than 1-wt% of retained austenite.

**Figure 7:**
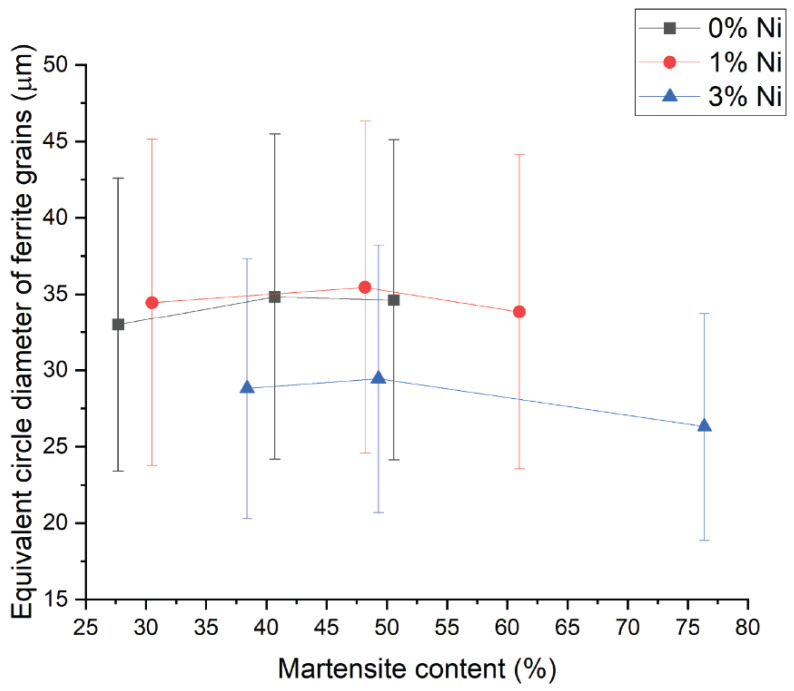
Ferrite grain size characterization by EBSD for varying content of martensite and Ni. Refining effect evident when increasing Ni content to 3-wt% Ni.

The ferrite grain size distribution in μm plotted in Figure 7 was obtained by correlation following the ASTM E112-13 standard [34]. An apparent reduction of the equivalent circle diameter was observed when comparing the 3-wt% Ni against the lower Ni contents.

### 3.3. Mechanical Characterisation

**Figure 8:**
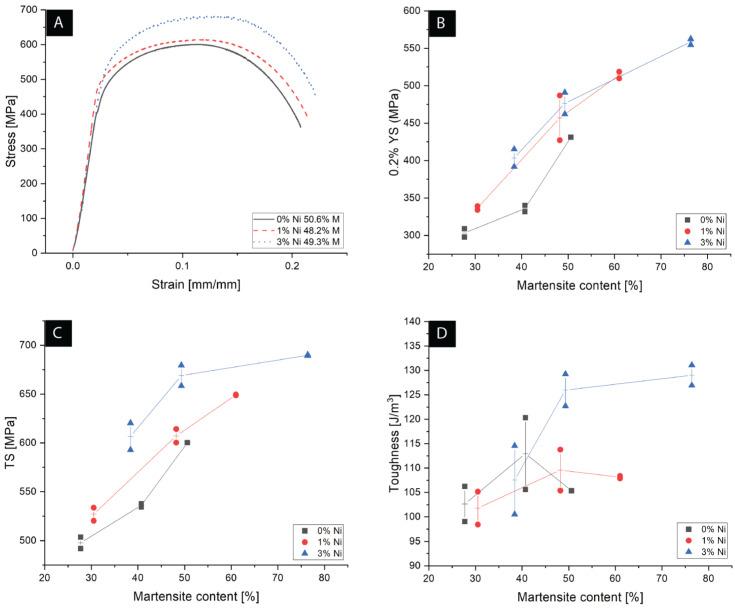
Representative engineering tensile stress–strain curves for martensite content around 50% with 0, 1, and 3-wt% Ni (**A**). Variation of 0.2% YS (**B**), TS (**C**), and toughness (**D**), as a function of the martensite and nickel content.

Representative engineering tensile stress–strain curves of 0, 1, and 3-wt% Ni DP-LASs with a martensite content of approximately 50% are summarized in Figure 8A. Additionally, the rest of the tensile curves are included in the Supplementary Figure S4. The absence of a yield point is characteristic of DP-LASs as the martensitic transformation during quenching results in unpinned dislocations in ferrite due to volume changes from austenite to martensite [35]. From the stress–strain curves, the calculated yield strength (0.2% YS) values shown in Figure 8B described an increasing trend with martensite content. In addition, greater 0.2% YS values were measured on samples with increasing Ni contents. The dependence of the tensile strength (TS) with the martensite volume as a function of Ni content is shown in Figure 8C, in which an increase in martensite volume increased TS. The same increasing behavior was observed with Ni content. Finally, as described in Figure 8D, toughness also increased for the highest nickel content compared to 0 and 1-wt% Ni when the martensite content exceeded 50%.

### 3.4. Fractography

**Figure 9:**
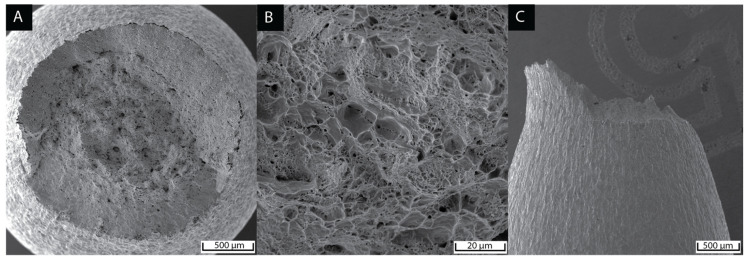
Top view of 0-wt% Ni fractured sample with 40.7% martensite content (**A**,**B**). Side view of the same sample (**C**).

All tensile test samples exhibited a ductile fracture with a cup-and-cone appearance as shown in Figure 9. A visible necking region was present regardless of the martensite and nickel content, indicating a large amount of plastic deformation prior to rupture. In addition, the fibrous fracture surfaces showed ductile features consisting of dimples usually attributed to nucleation, growth, and void coalescence. Microvoids were formed by the separation of the ferritic matrix from martensite or a harder second phase, such as carbides. The morphology of these voids was globular with smooth edges. Despite the controlled small amount of impurity elements in the research-grade alloys used in this work, the fracture surface showed pull-outs that could be related to decohesion at inclusion/matrix interfaces.

**Figure 10:**
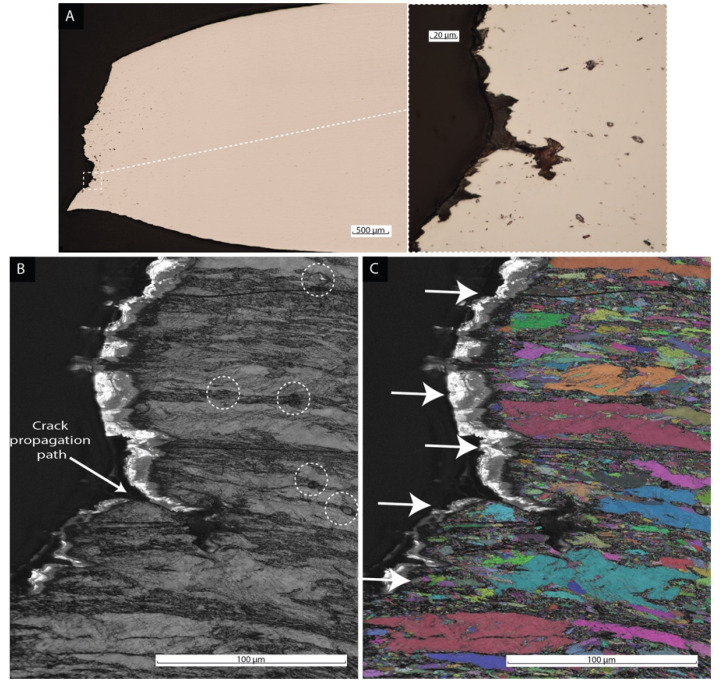
LOM cross-section of surface fracture from 0-wt% Ni 40.7% martensite content (**A**). Micrograph (**B**) represents an EBSD map with band contrast in which dark regions are martensite, whereas grey areas represent ferrite. An arrow indicates the crack propagation path through the martensite region, and microvoids are highlighted as dash circles. Micrograph (**C**) shows an EBSD mapping in which each grain is color coded with arrows indicating the martensitic bands.

The crack propagation morphology was studied by cross-section analysis of the fracture zone using the sample shown in Figure 9 (i.e., 0-wt% Ni 40.7% martensite content). The fractured sample was mounted in epoxy resin and reduced in thickness to approximately half the size. Next, the polished surface was characterized by light optical microscopy (LOM) to identify the region of interest, as shown in Figure 10A. Finally, the sample was ion milled and carbon-coated, following the EBSD procedure detailed in the Section 2. Two EBSD maps were selected for this analysis. Figure 10B represents a band contrast (BC) map, in which the dark regions indicate the martensitic phase and the grey zones the presence of ferrite. The arrow shows the crack propagation through the martensitic phase, and the dash circles depict microvoids limited to the martensite regions. Additionally, the map shown in Figure 10C represents each grain orientation with random colors to differentiate the grains in which the martensitic bands are indicated by arrows. An elongated grain distribution was observed due to the plastic deformation due to the tensile load in the necking zone.

## 4. Discussion

The as-received material had a microstructure composed of ferrite and pearlite. The banded morphology is often a consequence of through-thickness gradient in shear, applied deformation, and temperature [15]. Due to decarburization (i.e., a surface alteration phenomenon), the microstructure characterization in this work focused on the bulk, specifically, the center of the plates. Likewise, the surfaces of the samples used for mechanical testing were ground and polished prior to testing to remove artifacts that could be associated with a decarburized layer.

The presence of retained austenite represents a risk due to its potential transformation to untempered martensite, thus, having a deleterious effect on mechanical properties and HE resistance. Yoshino and Minozaki reported an increased SSC susceptibility when the untempered martensite content exceeded 5% in volume [13]. Since Ni additions promote retained austenite [7], the quantification of retained austenite was performed on a 3-wt% Ni sample. The diffraction pattern shown in Figure 6 suggested that the amount of retained austenite was below the 1-vol% detection threshold of the method.

Image analysis based on systematic manual point counting, following the ASTM E562-19 standard [27], was performed to correspond the phase ratio to the intercritical annealing holding temperature. The martensitic phase increased linearly with increasing holding temperature for a given Ni content, as shown in Figure 3. This behavior was expected based on the lever rule; as the austenite content increases with the intercritical temperature, more martensite will be introduced after the quenching transformation [36]. An increasing Ni content lowers A_c1_ and A_c3_ according to Equations (1) and (2) [25,35]. This effect was visible when fixing the intercritical annealing temperature in Figure 3 and comparing the martensite content as a function of Ni. For instance, with a holding temperature of 720 °C, the obtained martensite content was 15%, 28%, and 65% for 0, 1, and 3-wt% Ni, respectively.

Once the phase identification was completed, hardness testing was performed on the untempered samples. A larger martensite content affects the microstructure hardness in two ways. A direct consequence of the greater hardness of martensite compared to ferrite; therefore, a larger martensite volume fraction increases average macroscopic hardness. An indirect effect is associated with the carbon content of the martensitic phase, as the carbon content of martensite increases hardness. At a fixed global carbon content, increasing the martensite volume reduces the martensite carbon content, decreasing hardness [37]. As shown in Figure 4, in the untempered condition, increasing the martensite volume increased hardness for all Ni compositions, suggesting that the direct effect of martensite volume on microhardness was dominant. Although an increase of hardness with nickel content would be expected due to grain refinement and solid solution strengthening [7], when comparing the effect of Ni with the same martensite content for the untempered condition, no clear trend was observed. As the hardness values for most of the untempered samples were above the ASME B31.12 [3] and ISO 15156-2 [9] standards threshold, a tempering stage was necessary to meet the standard requirements.

The tempering stage reduced the hardness to a value below the required 250 HV threshold. The hardness reduction by tempering is linked to changes at the microstructural level. Firstly, carbide precipitation occurs as the carbon present in martensite is released, reducing the solid solution strengthening effect [33]. Secondly, dislocation recovery from the martensitic phase takes place [35]. To simplify the tempering evaluation, holding time was set as a variable, and the holding temperature was fixed at 550 °C (i.e., a common temperature used in commercial heat treatments). After tempering for 20 min, most samples’ hardness met the ASTM 31.12 and ISO 15156-2 standard except for the 3-wt% Ni DP_70% sample, which required a 60 min tempering step to reduce the hardness below the 250-HV limit. In this regard, the increase in tempering resistance caused by Ni was evident for the highest Ni content. When comparing the mean values, the reduction in hardness of the 3-wt% Ni samples after tempering was limited to 15%, whereas it varied between 35 and 45% for the 0 and 1-wt% Ni DP-LASs. In other words, the softening effect of tempering had a more significant impact on the lower Ni content samples. Further microstructure characterization was conducted to correlate the effect of tempering on hardness with its metallurgical alterations.

The effect of tempering on the microstructure was analyzed by SEM. Figure 5A shows the microstructure before tempering, composed of ferrite and martensitic islands. In some cases, carbides were visible within the ferritic grains, which could be linked to undissolved carbides from the ferritic–austenitic range [32]. The untempered martensite contained packets of individual units called laths with determined orientations [38]. Microstructural evolution due to tempering resulted in carbide precipitation, as shown in Figure 6B. A greater carbide size (150–400 nm) had preferential precipitation on prior lath boundaries and austenitic grain boundaries. A smaller carbide size distribution (30–150 nm) was also found between lath boundaries. Based on the choice of tempering temperature (i.e., 550 °C), the observed carbides are expected to be cementite [39].

As part of the microstructural characterization, grain size distribution was evaluated for each microstructure ratio and Ni composition. The analysis performed by EBSD was centered on the ferrite grains as the indexation on the martensitic phase is difficult due to their low diffraction pattern quality. The decrease in the mean grain size with increasing Ni content from 1 to 3% shown in Figure 7 can be explained by nickel’s refining effect on the microstructure [7]. Kim et al. [40] reported a similar grain size reduction when increasing Ni from 0 to 2 wt-% content on 0.3-wt% C-1.5-wt% Mn-1.5-wt% Si steels.

As seen in Figure 8A, the engineering stress–strain curves showed a continuous yielding, typical in DP ferritic–martensitic steels [15,37,41]. Continuous yielding is linked to unpinned dislocations in ferrite, introduced in the austenite to martensite transformation during the material processing [35]. Larger martensite content increased yield and tensile strength, as summarized in Figure 8B,C, respectively. These findings match Marder [42] and Davies [43] observations, which indicated that YS and TS increase linearly with martensite volume but are independent of the martensite carbon content. From a composite perspective, the increased amount of hard particles in a soft phase produces a hardening effect according to the rule of mixture [35]. For the three microstructure ratios, an increase in Ni content produced an increase in YS and TS. The increase in strength was attributed to solid solution strengthening and grain refinement [7,35]. The toughness results presented in Figure 8D were estimated from the engineering stress–strain curves by integration (i.e., the area under the curve) for a qualitative comparison. Samples with a low martensite content (<50%) exhibited no evident effect of Ni, plausibly due to the combined effects in elongation to failure, YS, and TS, which influence the area under the stress–strain curves. Conversely, substantially higher toughness values were observed for the 3-wt% Ni at ≥50% martensite. This difference in toughness could was linked to the combined effect of grain refinement and increased YS and TS due to Ni additions [7].

The fracture surface exhibited a typical ductile appearance of cup-and-cone. The necking deformation observed by SEM in Figure 9 matched the deformation after ultimate tensile strength in the engineering stress–strain curves. The fracture mechanism was linked to the nucleation, growth, and coalescence of microvoids mainly located in the martensitic regions. The density of these microvoids was more extensive in the necking zone, as detected by LOM in Figure 10A. Their larger size is due to microvoids coalescence in the necking region. The microvoid distribution was associated with the martensite band distribution in agreement with Mazinani et al. [44] An evaluation of the crack propagation on a fractured sample cross-section by EBSD (Figure 10B,C) showed that the crack evolution path followed the banded martensite phase. As reported by Lacroix et al., damage sites were linked to martensite grains, such as decohesion between martensite grains [45].

The results presented herein suggest that the 3-wt% DP-LAS with a 50% martensite might represent the optimal microstructure conditions, as it combines high strength and toughness with a hardness that meets the ISO 15156-2 and ASME B31.12 limit [3,9]. Nevertheless, the influence of the DP microstructure of HE needs to be investigated. The procedures and DP-LASs developed herein will be used in subsequent investigations to quantify hydrogen transport kinetics and hydrogen embrittlement resistance. The final objective of the program is to determine the role of microstructure and nickel on environmentally assisted cracking and the potential to introduce DP microstructures in hydrogen-related applications.

## 5. Conclusions

This study correlated the intercritical annealing temperatures and martensite content of research-grade low-alloy steels with varying Ni content. In addition, tempering variables were designed to meet the ASME B13.12 and ISO 15156-2 hardness limitations. Further, the effect of microstructure and Ni content on the mechanical properties of ferritic–martensitic DP-LAS was analyzed.

- Except for the 3-wt% Ni case, tempering for 20 min at 550 °C was enough to meet the ASME B31.12 and ISO 15156-2 standard hardness limitation (250-HV). The softening effect obtained after tempering was observed by SEM, revealing fine carbide precipitation.
- XRD showed the absence of retained austenite after tempering. In addition, the refinement effect of Ni on the ferritic grain size was evident, as quantified by EBSD analysis.
- 0.2%YS and TS increased with martensite fraction. Similarly, an evident increase in TS was found with increasing Ni content.
- The 3-wt% Ni with a martensite content exceeding 50% had the highest toughness values, which can be linked to the high 0.2% YS and TS.
- Samples exhibited a ductile fracture with a cup-and-cone appearance. The fracture mechanism was linked to the coalescence of microvoids and decohesion between martensitic grains.
- The 3-wt% DP-LAS with a 50% martensite was the optimal microstructure condition, as it combines high strength and toughness with a hardness that meet the ASME B31.12 and ISO 15156-2 standard limit.

Future work is needed to evaluate the effect of the DP microstructure on the HE resistance. Further analysis is also required to determine the potential commercial scalability of the heat treatment process to ensure a uniform microstructure through the thickness.

## Figures and Tables

**Table 1:** Chemical composition of LAS obtained following ASTM E1019-11/CO [23] and ASTM E1479-99/CTP 3101/ICP [24].

Alloy	Ni (wt%)	Mn (wt%)	Si (wt%)	C (wt%)
**0-wt% Ni**	0.00	1.30	0.24	0.17
**1-wt% Ni**	0.97	1.30	0.24	0.17
**3-wt% Ni**	2.86	1.30	0.24	0.17

## Data Availability

Not applicable.

## Supplementary Materials

The following supporting information can be downloaded at: https://www.mdpi.com/article/10.3390/ma15249018/s1.

## References

[1] Dwivedi S.K., Vishwakarma M. (2018). Hydrogen embrittlement in different materials: A review. Int. J. Hydrogen Energy.

[2] Baykara S.Z. (2018). Hydrogen: A brief overview on its sources, production and environmental impact. Int. J. Hydrogen Energy.

[3] (2019). Hydrogen Piping and Pipelines.

[4] Fekete J.R., Sowards J.W., Amaro R.L. (2015). Economic impact of applying high strength steels in hydrogen gas pipelines. Int. J. Hydrogen Energy.

[5] (2021). Standard Terminology and Acronyms Relating to Corrosion.

[6] Snape E. (1967). Sulfide Stress Corrosion of Some Medium and Low Alloy Steels. Corrosion.

[7] Kappes M., Iannuzzi M., Rebak R.B., Carranza R.M. (2014). Sulfide stress cracking of nickel-containing low-alloy steels. Corros. Rev..

[8] Craig B.D. (1982). The effect of nickel on hydrogen cracking resistance in low alloy steels—A review. Corrosion.

[9] (2020). Petroleum and Natural Gas Industries—Materials for Use in H_2_S-Containing Environments in Oil and Gas Production—Part 2: Cracking-Resistant Carbon and Low-Alloy Steels, and the Use of Cast Irons.

[10] Treseder R.S., Swanson T.M. (1968). Factors in sulfide corrosion cracking of high strength steels. Corrosion.

[11] Snape E. (1968). Roles of composition and microstructure in sulfide cracking of steel. Corrosion.

[12] Payer J., Pednekar S., Boyd W. (1986). Sulfide stress cracking susceptibility of nickel containing steels. Metall. Trans. A.

[13] Yoshino Y., Minozaki Y. (1986). Sulfide stress cracking resistance of low-alloy nickel steels. Corrosion.

[14] Biagiotti S.F., Matlock D.K., Krauss G. (1995). Effect of Ni Content and Tempering Temperature on SSC Resistance in Modified 4340 Steels.

[15] Tasan C.C., Diehl M., Yan D., Bechtold M., Roters F., Schemmann L., Zheng C., Peranio N., Ponge D., Koyama M. (2015). An Overview of Dual-Phase Steels: Advances in Microstructure-Oriented Processing and Micromechanically Guided Design. Annu. Rev. Mater. Res..

[16] Snape E., Schaller R.F., Forbes Jones R.M. (1969). A method for improving hydrogen sulfide accelerated cracking resistance of low alloy steels. Corrosion.

[17] Davies R.G. (1981). Hydrogen embrittlement of dual-phase steels. Metall. Trans. A.

[18] Koyama M., Tasan C.C., Akiyama E., Tsuzaki K., Raabe D. (2014). Hydrogen-assisted decohesion and localized plasticity in dual-phase steel. Acta Mater..

[19] Liu Q., Zhou Q., Venezuela J., Zhang M., Atrens A. (2018). The role of the microstructure on the influence of hydrogen on some advanced high-strength steels. Mater. Sci. Eng. A.

[20] Sun S., Gu J., Chen N. (1989). The influence of hydrogen on the sub-structure of the martensite and ferrite dual-phase steel. Scr. Metall..

[21] Alp T., Iskanderani F.I., Zahed A.H. (1991). Hydrogen effects in a dual-phase microalloy steel. J. Mater. Sci..

[22] Chan S.L.I. (1999). Hydrogen trapping ability of steels with different microstructures. J. Chin. Inst. Eng..

[23] (2011). Standard Test Methods for Determination of Carbon, Sulfur, Nitrogen, and Oxygen in Steel, Iron, Nickel, and Cobalt Alloys by Various Combustion and Fusion Techniques.

[24] (2011). Standard Practice for Describing and Specifying Inductively-Coupled Plasma Atomic Emission Spectrometers.

[25] Andrews K.W. (1965). Empirical formulae for the calculation of some transformation temperatures. J. Iron. Steel. Inst..

[26] Parodi S. (2018). Obtaining Low Alloy Steels with Different Ni Contents and Similar Microstructures and Mechanical Properties.

[27] (2019). Standard Test Method for Determining Volume Fraction by Systematic Manual Point Count.

[28] (2019). Standard Practice for Determining Average Grain Size Using Electron Backscatter Diffraction (EBSD) in Fully Recrystallized Polycrystalline Materials.

[29] (2017). Standard Test Method for Microindentation Hardness of Materials.

[30] (2021). Standard Test Methods for Tension Testing of Metallic Materials.

[31] Liu A.F. (2005). Mechanics and Mechanisms of Fracture: An Introduction.

[32] Kuang C., Li J., Zhang S., Wang J., Liu H., Volinsky A. (2014). Effects of quenching and tempering on the microstructure and bake hardening behavior of ferrite and dual phase steels. Mater. Sci. Eng. A.

[33] Hosford W.F. (2010). Mechanical Behavior of Materials.

[34] (2021). Standard Test Methods for Determining Average Grain Size.

[35] Krauss G. (2015). Steels: Processing, Structure, and Performance.

[36] Callister W.D. (2000). Fundamentals of Materials Science and Engineering.

[37] Movahed P., Kolahgar S., Marashi S.P.H., Pouranvari M., Parvin N. (2009). The effect of intercritical heat treatment temperature on the tensile properties and work hardening behavior of ferrite–martensite dual phase steel sheets. Mater. Sci. Eng. A.

[38] Krauss G., Marder A. (1971). The morphology of martensite in iron alloys. Metall. Trans..

[39] Handbook A.S.M.M. (1991). Heat Treatment.

[40] Kim K., Lee S.-J. (2017). Effect of Ni addition on the mechanical behavior of quenching and partitioning (Q&P) steel. Mater. Sci. Eng. A.

[41] Ghaheri A., Shafyei A., Honarmand M. (2014). Effects of inter-critical temperatures on martensite morphology, volume fraction and mechanical properties of dual-phase steels obtained from direct and continuous annealing cycles. Mater. Des. (1980–2015).

[42] Marder A.R. (1982). Deformation characteristics of dual-phase steels. Metall. Trans. A.

[43] Davies R.G. (1978). Influence of martensite composition and content on the properties of dual phase steels. Metall. Trans. A.

[44] Mazinani M., Poole W.J. (2007). Effect of martensite plasticity on the deformation behavior of a low-carbon dual-phase steel. Metall. Mater. Trans. A.

[45] Lacroix G., Pardoen T., Jacques P.J. (2008). The fracture toughness of TRIP-assisted multiphase steels. Acta Mater..

