# Supplementary material for: Effect of Intercritical Temperature on the Microstructure and Mechanical Properties of a Ferritic–Martensitic Dual-Phase Low-Alloy Steel with Varying Nickel Content
Source: Materials (Basel). 2022 Dec 16;15(24):9018. doi: 10.3390/ma15249018 (PMC9788566; doi:10.3390/ma15249018)
Supplement: Supplementary file 1 [file materials-15-09018-s001.zip › materials-2071180-supplementary.pdf]

materials

MDPI

Supplementary Materials

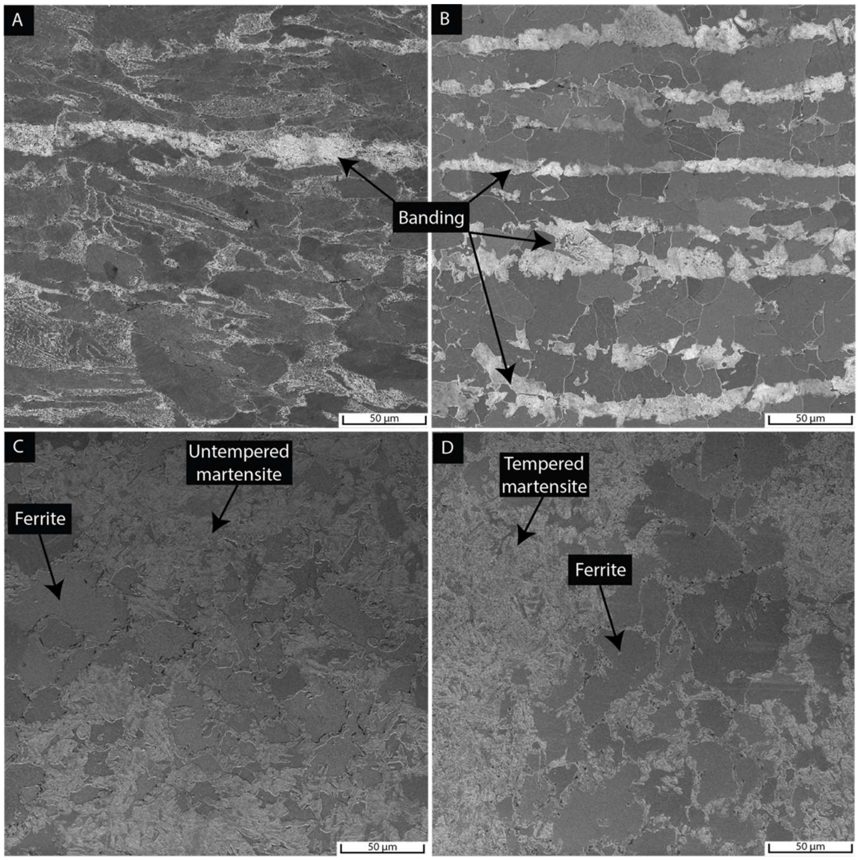

Supplementary Figure S1. SEM images of the heat-treated samples at different heat treatment stages for sample 1-wt% Ni DP_70%. Micrographs represent the (**A**) As-received, (**B**) Annealed, (**C**) As-quenched, and (**D**) Tempered conditions.

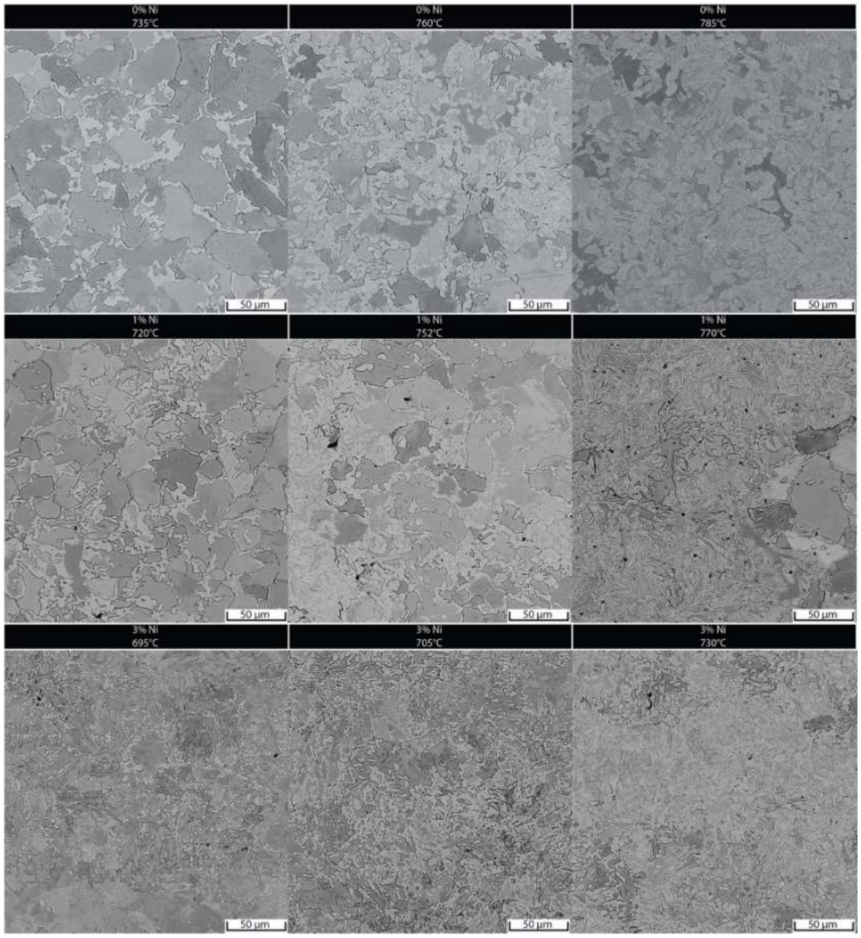

Supplementary Figure S2. SEM images of samples with varying Ni content and intercritical annealing temperatures. Increasing martensite content with the increasing temperature.

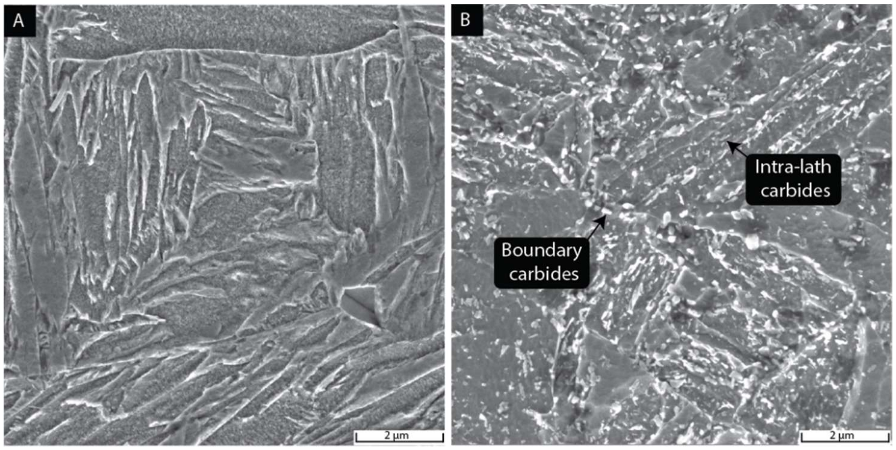

Supplementary Figure S3. SEM images of 1-wt% Ni DP_70% sample in the as-quenched condition (**A**) and after temper (**B**). Untempered martensite displays a needle and rough appearance while ferrite is present as a smooth area. Tempered microstructure displayed carbides at boundaries and within martensitic laths.

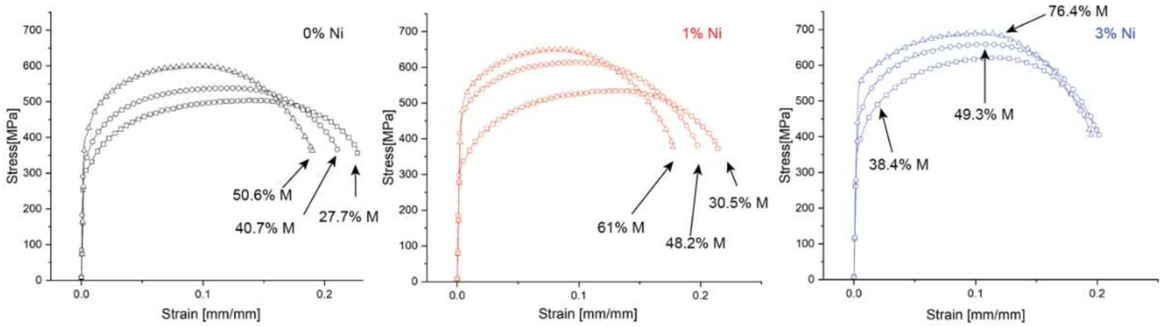

Supplementary Figure S4. Engineering tensile curves for varying Ni and martensite contents.
